# Role of serum amyloid A in Mycoplasma pneumoniae pneumonia and related respiratory diseases

**DOI:** 10.3389/fped.2026.1902333

**Published:** 2026-09-07

**Authors:** Fengqin Zhang, Yingqian Zhang

**Affiliations:** 1 Graduate School, Hebei North University, Zhangjiakou, China; 2 Department of Respiratory Medicine, Hebei Children's Hospital, Shijiazhuang, Hebei Province, China; 3 Hebei Clinical Medicine Research Center for Children's Health and Diseases, Hebei Children's Hospital, Shijiazhuang, Hebei Province, China; 4 Hebei Key Laboratory of Pediatric Allergy and Immunology, Hebei Children's Hospital, Shijiazhuang, Hebei Province, China

**Keywords:** acute respiratory distress syndrome, CARDS toxin, FPR2, HDL remodeling, Mycoplasma pneumoniae pneumonia, NLRP3 inflammasome, refractory MPP, serum amyloid A

## Abstract

Serum amyloid A (SAA) is an acute-phase protein that can increase in plasma concentration by up to 1000-fold during infection and inflammation. Clinical studies have identified significantly elevated SAA levels in *Mycoplasma pneumoniae* pneumonia (MPP), correlating with disease severity, lung injury, and prognosis. Emerging evidence indicates that SAA is not merely a passive inflammatory biomarker but an active contributor to MPP pathogenesis, which engages receptors such as formyl peptide receptor 2 (FPR2), Toll-like receptor 2 (TLR2), Toll-like receptor 4 (TLR4), and the receptor for advanced glycation end products (RAGE) to intensify the inflammatory response. The proposed Community-Acquired Respiratory Distress Syndrome (CARDS) toxin-NLR family pyrin domain containing 3 (NLRP3)-Interleukin-1*β* (IL-1*β*)-SAA amplification loop synthesizes evidence from disparate experimental systems into a coherent mechanistic hypothesis; however, this pathway has not been experimentally verified in a single integrated model, and each component has been demonstrated independently rather than as a functional loop. SAA has demonstrated diagnostic and predictive value in MPP, with reported area under the curve(AUC) values of 0.942 for MPP diagnosis and 0.735 -0.872 for predicting refractory MPP(RMPP) across studies with variable designs; however, detailed performance metrics, including 95% confidence intervals (CIs), study populations, and sample sizes are limited by inconsistent reporting in the source literature. SAA is induced within 4–6 hours post-infection, more rapidly than C-reactive protein (CRP), procalcitonin (PCT), and interleukin-6 (IL-6); however, its limited specificity requires interpretation alongside clinical findings and other biomarkers. Moreover, SAA plays context-dependent roles in various pulmonary diseases, such as chronic airway diseases, interstitial lung diseases, acute infectious pneumonias, and acute lung injury. The dual nature of SAA—protective at low levels but pathogenic when persistently elevated—presents a therapeutic challenge: balancing acute-phase host defense with the prevention of chronic inflammatory damage. This review consolidates current knowledge on SAA structural biology, high-density lipoprotein (HDL)-associated regulation, receptor-mediated signaling, and clinical applications in MPP. It also examines SAA roles across pulmonary diseases, cellular sources, mechanisms of action, and emerging therapeutic strategies targeting SAA-mediated pathways.

## 1 Introduction

*Mycoplasma pneumoniae* pneumonia (MPP) presents a significant clinical challenge, particularly in children. *M. pneumoniae* is a leading cause of community-acquired pneumonia (CAP) in children and adolescents, accounting for 10-40% of cases (1–3). While most MPP cases are mild and self-limiting, approximately 10-30% progress to refractory MPP (RMPP) or severe *Mycoplasma pneumoniae* pneumonia (SMPP). These advanced stages are characterized by persistent fever, worsening radiographic findings, and extrapulmonary complications despite appropriate antibiotic treatment (4, 5). Macrolide resistance rates of 69–95% have been reported predominantly in pediatric cohorts from China and other East Asian regions, However, rates vary according to geographic location, study period, and population characteristics, complicating treatment strategies. This underscores the urgent need to understand the immunopathological mechanisms driving disease progression (6, 7, 30).A key feature of *M. pneumoniae* pathogenesis is the production of the Community-Acquired Respiratory Distress Syndrome (CARDS) toxin. a unique ADP-ribosylating and vacuolating toxin that interacts specifically with surfactant protein A and annexin A2 on the host airway epithelial cells (8, 9). Once internalized, the CARDS toxin activates the NLR family pyrin domain containing 3 (NLRP3) inflammasome, leading to the caspase-1-dependent maturation and release of interleukin-1*β* (IL-1*β*) and interleukin-18 (IL-18) (10, 11). This CARDS toxin-NLRP3-IL-1*β* pathway represents a pathogen-specific immunopathological mechanism that distinguishes MPP from viral pneumonias and other bacterial respiratory infections.

Among the key mediators of this immunopathological cascade, serum amyloid A (SAA) plays a particularly important role. This acute-phase protein family in humans includes four members: SAA1 and SAA2, primarily produced by the liver as acute-phase reactants; SAA3, a pseudogene; and SAA4, which is consistently expressed (12, 13). Normally, SAA levels in the bloodstream are low (<10 mg/L). However, during infection, these levels can surge up to 1000-fold within 4-6 hours, preceding the rise of C-reactive protein (CRP) by 24-48 hours (14, 15). In the context of MPP, evidence increasingly indicates that SAA acts not just as a byproduct of inflammation but as a significant contributor to disease pathogenesis. It enhances neutrophil recruitment, promotes the formation of neutrophil extracellular traps (NETs), facilitates T helper 17 (Th17) differentiation, and perpetuates an inflammatory loop through various receptor-mediated pathways (16–18).

An important advance in SAA biology has been the recognition that SAA functions as more than a passive biomarker. The high-density lipoprotein (HDL)-shielding hypothesis, derived largely from *in vitro* biochemical experiments and animal model data, suggests that, under normal physiological conditions, most circulating SAA is bound to HDL, which masks its pro-inflammatory characteristics (19, 20). During severe inflammation, SAA displaces apolipoprotein A-I(Apo A-I) from HDL, resulting in the release of free SAA. This release is facilitated by cholesteryl ester transfer protein (CETP)-mediated remodeling, proteolytic cleavage, and oxidative modification, which activate the pathogenic potential of SAA (21, 22).The dual role of SAA—protective at low concentrations through opsonization and tissue repair, yet pathogenic at high levels via receptor-mediated inflammatory amplification—poses a significant therapeutic challenge that extends beyond metabolic syndrome to a broader range of inflammatory diseases.

This review offers a comprehensive synthesis of SAA biology across eight interconnected domains: structural biology and synthesis regulation; HDL-associated shielding and activation; receptor-mediated signaling mechanisms; clinical applications inMPP; comparative roles in various pulmonary diseases; therapeutic strategies; cellular sources and modes of action; and future research directions. The literature search was conducted using PubMed, Web of Science, Embase, and CNKI databases with combinations of keywords including “*Mycoplasma pneumoniae* pneumonia,” “MPP,” “SAA,” “serum amyloid A,” “inflammation,” “biomarkers,” “NLRP3,” “IL-1*β*,” and “CARDS toxin.” Articles were considered eligible if they evaluated SAA-related mechanisms, clinical associations, diagnostic performance, or therapeutic implications in MPP or related respiratory infections. Studies not directly related to MPP or SAA biology, conference abstracts without sufficient primary data, and duplicate publications were excluded. Only articles published in English or Chinese were included, covering the publication period from 2000 to 2025. This integrated framework aims to link current mechanistic insights with clinical applications and identify the most promising avenues for therapeutic intervention.

## 2 Structural biology and synthesis regulation of SAA

### 2.1 Regulation of SAA production: Il-6/Stat3 axis and hepatic/local synthesis

**Figure 1:**
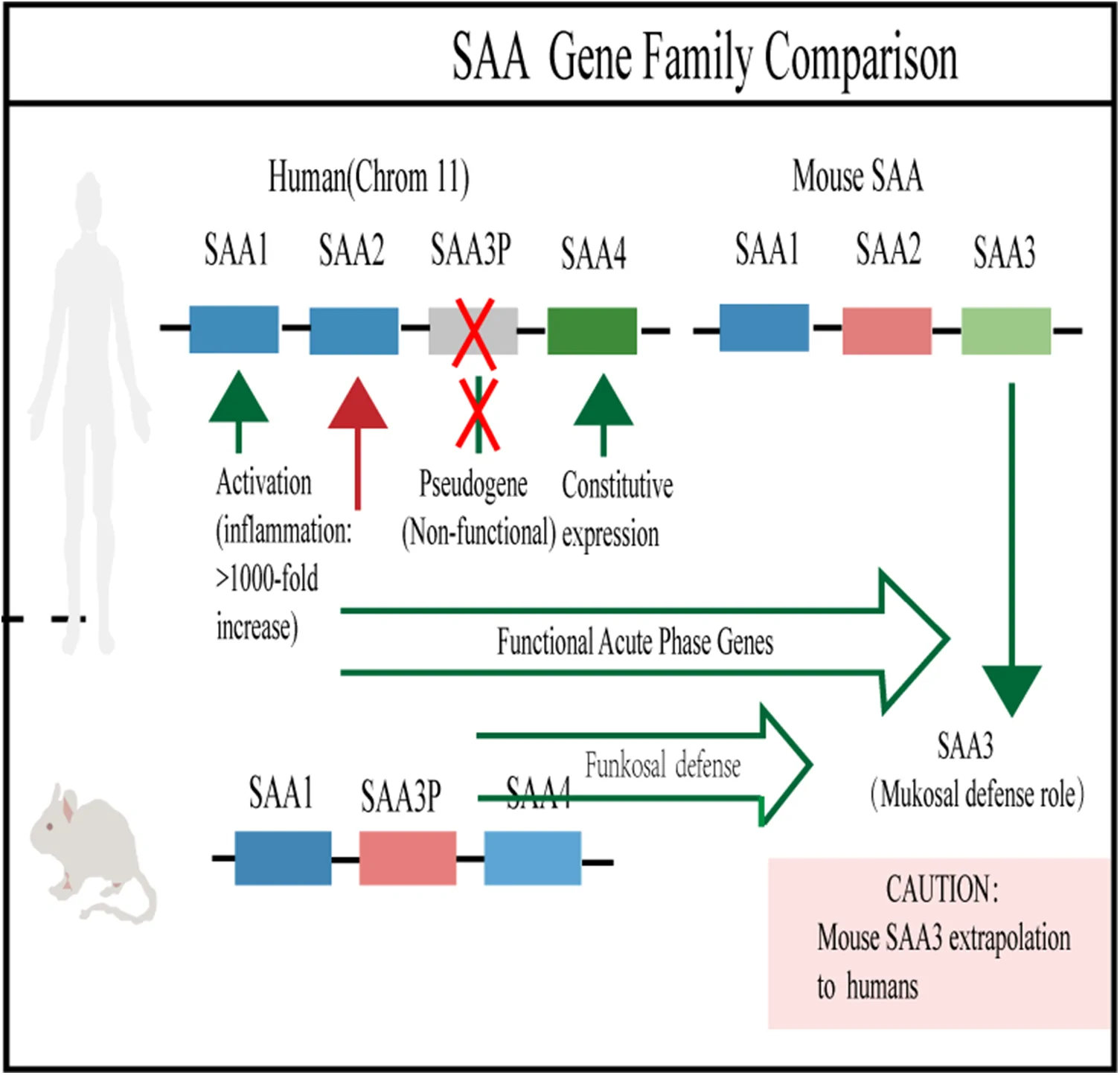
The SAA gene family in humans and mice. **(A)** Human SAA genes are located on chromosome 11p15.1. SAA1 and SAA2 encode the major acute-phase isoforms, SAA3 is a pseudogene, and SAA4 is constitutively expressed. **(B)** Murine Saa genes are located on chromosome 7. SAA1, SAA2, and SAA3 all encode acute-phase isoforms, while Saa4 encodes constitutively expressed SAA. Approximate gene sizes and intergenic distances are indicated. This figure was created based on data from Sack (12) and Buck et al. (13).

SAA synthesis is mainly controlled by pro-inflammatory cytokines through the IL-6/STAT3 signaling pathway. After *M. pneumoniae* infection, pattern recognition receptors (PRRs) such as Toll-like receptor 2 (TLR2), Toll-like receptor 4 (TLR4), and Ficolin-3 detect pathogen-associated molecular patterns (PAMPs), triggering nuclear factor kappa B (NF-*κ*B) activation. This activation results in the production of interleukin-1*β* (IL-1*β*), interleukin-6 (IL-6), and tumor necrosis factor-alpha (TNF-*α*) (23–25). Among these cytokines, IL-6 is the primary inducer, activating the signal transducer and activator of transcription 3 (STAT3) transcription factor in hepatocytes. This activation leads to the strong expression of the SAA1 and SAA2 genes via STAT3 response elements in the SAA promoter region (26, 27). Furthermore, transcription factors such as NF-*κ*B, CCAAT/enhancer-binding protein *β* (C/EBP*β*), and activator protein 1 (AP-1) contribute to the transcriptional regulation of SAA, forming a complex regulatory network that supports the rapid and significant induction typical of the acute-phase response. (Figure 1).

Recent studies indicate that SAA production extends beyond the liver. In MPP, bronchial epithelial cells and alveolar macrophages locally synthesize SAA when directly stimulated by *M. pneumoniae*. This process creates a paracrine/autocrine amplification loop, which may be particularly relevant in severe cases of the disease (28, 29).This localized SAA production may explain why SAA levels in bronchoalveolar lavage fluid (BALF) correlate more strongly with lung injury severity than do systemic SAA levels (16).

The CARDS toxin is proposed to play a role in inducing SAA production specific to MPP. Once internalized, the CARDS toxin triggers the assembly of the NLRP3 inflammasome, leading to the formation of ASC specks and activation of caspase-1. This process results in the maturation and release of IL-1*β* and IL-18 (8, 10). IL-1*β*, in turn, serves as a potent inducer of SAA gene expression, creating a “CARDS toxin-NLRP3-IL-1*β*-SAA” amplification loop (11). However, each step of this proposed loop has been demonstrated in separate experimental systems. and the complete CARDS toxin-NLRP3-IL-1*β*-SAA amplification loop has yet to be experimentally verified in a single model or in human MPP.

Recent studies have highlighted the role of long non-coding RNAs in regulating SAA. Notably, lncRNA MALAT1 influences macrophage polarization and progression by modulating NF-*κ*B signaling. *M. pneumoniae* infection activates NF-*κ* B signaling, as shown by the phosphorylation and nuclear translocation of the p65 subunit. Additionally, research shows that knocking down MALAT1 significantly inhibits NF-*κ*B activation triggered by *M. pneumoniae* infection, indicating a potential regulatory mechanism upstream of SAA production (31). Given that NF-*κ*B cooperates with STAT3 and C/EBP*β* to drive hepatic SAA1 and SAA2 gene transcription (Section 2.1), MALAT1-mediated NF-*κ*B modulation represents a proposed upstream regulatory node linking M. pneumoniae infection to hepatic acute-phase SAA synthesis.

#### 2.1.2 Evidence status of the cards toxin-NLRP3-Il-1*β*-SAA amplification loop

The CARDS toxin-NLRP3-IL-1*β*-SAA amplification loop proposed in this review integrates findings from multiple experimental systems into a coherent mechanistic framework. However, it is important to distinguish which components of this loop are experimentally established and which remain hypothetical (Table 1).

**Table 1:** Evidence status of the proposed CARDS toxin-NLRP3-IL-1*β*-SAA amplification loop in Mycoplasma pneumoniae pneumonia.

Component	Evidence	Key references	Knowledge gaps
CARDS toxin → NLRP3 activation	Established in human airway epithelial cells and murine models	(8–11)	Cell-type specificity in intact lung tissue; role of co-receptors beyond SP-A and annexin A2
NLRP3→ IL-1*β*/IL-18 maturation	Established across multiple cell types and species	(10, 11)	Kinetics of IL-1β vs. IL-18 release; contribution of non-canonical inflammasome pathways
IL-1β → SAA induction	Established for hepatic SAA1/SAA2; local pulmonary production less characterized	(26, 27)	Relative contribution of IL-1β vs. IL-6 and TNF-*α* in acute vs. sustained SAA responses
SAA → TLR4/NLRP3 re-activation	Established in THP-1 cells and murine macrophages; not demonstrated in MPP-specific models	(55, 58, 59)	Concentration threshold for feedback activation; role of HDL-bound vs. free SAA
Complete loop integration	Not validated in any single experimental system	—	Integrated *in vivo* or *ex vivo* MPP models; human longitudinal biomarker studies

This table summarizes the evidence supporting each step of the proposed amplification loop and identifies critical gaps that limit its validation as an established pathogenic mechanism in MPP.

Established elements include: (i) CARDS toxin binding to SP-A and annexin A2 on airway epithelial cells and subsequent internalization (8, 9); (ii) CARDS toxin-mediated NLRP3 inflammasome activation with caspase-1-dependent maturation of IL-1*β* and IL-18 (10, 11); (iii) IL-1*β* as a potent inducer of hepatic SAA1 and SAA2 gene expression via NF-*κ*B and C/EBP*β* pathways (26, 27); and (iv) SAA engagement of TLR4/MD-2 leading to NF-*κ*B activation and secondary NLRP3 priming (55, 58, 59). Each of these steps has been independently validated in cell-based or animal models, though not always in the specific context of *M. pneumoniae* infection.

The critical gap lies in integration: no single experimental system—whether *in vitro*, *ex vivo*, or *in vivo*—has demonstrated the complete loop from CARDS toxin exposure to SAA-mediated feedback onto NLRP3. Specifically, whether SAA produced in response to CARDS toxin-induced IL-1*β* subsequently amplifies NLRP3 activity in pulmonary macrophages during MPP remains untested. The temporal dynamics of this proposed feedback (hours vs. days), the relative contribution of hepatic versus locally produced SAA, and the point at which this loop becomes self-sustaining rather than self-limiting are unknown.

This proposed loop should therefore be regarded as a working model that synthesizes available evidence and generates testable hypotheses, rather than an established pathogenic mechanism. Its predictive value for MPP severity, refractoriness, or therapeutic response requires validation in integrated models that simultaneously track CARDS toxin levels, inflammasome activity, IL-1*β*, and SAA in the same biological system.

### 2.2 Current challenges and controversies in the study of SAA

These methodological challenges directly constrain our ability to validate or refute mechanistic hypotheses, including the proposed CARDS toxin-NLRP3-IL-1*β*-SAA amplification loop discussed in Section 2.1.2.

Despite significant advancements in understanding SAA biology, several fundamental challenges remain. A major issue is that most *in vitro* studies rely on recombinant SAA proteins, which may not accurately reflect endogenous SAA due to differences in post-translational modifications, aggregation state, and lipidation status (32). Furthermore, recombinant SAA preparations often contain bacterial contaminants like lipopolysaccharide (LPS) and lipoproteins, which can skew results, and the bioactivity of these preparations varies across commercial sources.

SAA exists in several isoforms, including SAA1.1, SAA1.2, SAA1.3, SAA2.1, and SAA2.2, yet most clinical tests measure only total SAA without distinguishing between these subtypes (33). Recent research suggests that each subtype has distinct functions: for instance, SAA1.1 may have stronger pro-inflammatory effects than SAA1.3, while SAA2 variants might differ in their HDL binding affinity. This lack of differentiation in clinical practice limits the precision of SAA-related diagnostics and therapeutics.

Third, SAA interacts with multiple receptors, including FPR2, TLR2, TLR4, RAGE, SR-BI, and P2X7, either simultaneously or in sequence. The role each receptor plays varies depending on the cell type, disease context, and SAA concentration (34, 35). This receptor pleiotropy makes it difficult to assign specific functions to individual receptor pathways and complicates the development of receptor-selective therapeutic agents.

Fourth, the measurement of SAA lacks standardization. Different commercial assays (including enzyme-linked immunosorbent assay [ELISA], immunonephelometry, latex-enhanced immunoturbidimetric assays, and gold-labeled quantitative immunoassay platforms) use various antibodies and calibration materials, leading to significant variability in absolute SAA values across assays (36). Additionally, reported cutoffs for elevated SAA levels range widely from 10 mg/L to 100 mg/L, complicating cross-study comparisons. To effectively translate SAA research into clinical practice, establishing international reference standards and disease-specific reference ranges is essential.

A fundamental question remains unresolved: at what concentration and under conditions does SAA transition from a protective acute-phase protein to a pathogenic mediator? This concentration-dependent shift likely involves various factors, such as the initiating pathogen, host genetic background, comorbidities, and the kinetics of SAA induction and clearance. Addressing this issue will necessitate integrated approaches, including standardized assays, subtype-specific measurements, and rigorously controlled *in vivo* models.

## 3 HDL association and SAA activation

Under physiological conditions, most circulating SAA is associated with HDL particles, primarily binding to ApoA-I, the main structural protein of HDL (37). In mouse models, SAA1 and SAA2 have been shown to rapidly replace ApoA-I on HDL particles, and studies of human acute-phase HDL confirm significantly altered composition and function (37, 39). In fact, SAA can constitute up to 80% of the protein mass in acute-phase HDL, compared to less than 1% in normal HDL (37).

HDL remodeling significantly impacts its function. When HDL is enriched with SAA, its ability to facilitate cholesterol efflux and its antioxidant activity are reduced (38). In cell co-culture studies, this reduction has been attributed partly to SAA displacing paraoxonase-1 and platelet-activating factor acetylhydrolase, and clinical data confirm that HDL may acquire proinflammatory properties during the acute-phase response (40, 41). In a clinical study of children with M. pneumoniae infection, elevated SAA levels, which reflect increased SAA loading on HDL, are associated with greater disease severity, suggesting that SAA-HDL interactions might influence pulmonary inflammation (42). This impaired cholesterol efflux may be particularly consequential in the lungs, where surfactant lipid homeostasis is thought to depend in part on HDL-mediated lipid transport (45). However, direct evidence linking SAA-mediated HDL dysfunction to surfactant abnormalities in MPP is currently lacking.

The release of SAA from HDL is an important step in its pathogenic activation. At sites of inflammation, including the pulmonary microenvironment in MPP, several mechanisms are proposed to facilitate this release: (1) CETP-mediated lipid exchange among lipoproteins, which alters HDL structure and weakens SAA binding, as demonstrated in clinical studies of patients who underwent surgery (43); (2) proteolytic cleavage, which may involve neutrophil-derived elastase and cathepsin G at sites of inflammation; and (3) oxidative modification of HDL by reactive oxygen species generated during the neutrophil oxidative burst, as shown in *in vitro* biochemical analyses (44). Once SAA is liberated from the inhibitory effects of HDL, it can interact with cell-surface receptors and initiate proinflammatory responses.

The concept of HDL acting as a “shield” that limits the proinflammatory effects of SAA has been proposed as a coherent explanatory framework for the dual behavior of SAA. According to this hypothesis, SAA bound to HDL is predicted to have reduced capacity to activate cell-surface receptors compared to free SAA. During severe inflammatory conditions such as refractory MPP, it is hypothesized that the balance may shift toward free SAA, thereby permitting its pathogenic activities. However, direct experimental demonstration of this shielding mechanism in MPP is currently lacking. This framework draws indirect support from observations in other inflammatory conditions: for instance, in patients with obesity and asthma, elevated SAA-HDL levels are associated with monocyte secretion of IL-6, IL-1*β*, and TNF-*α* through the FPR2/ATP/P2X7 pathway, exacerbating allergen-induced neutrophilic airway inflammation (46). Whether a similar SAA-HDL-dependent mechanism operates in MPP remains to be determined.

It should be noted that direct experimental evidence for SAA-HDL binding dynamics, SAA release from HDL, and free versus HDL-bound SAA ratios specifically in MPP is currently limited. Much of the mechanistic framework discussed in this section is derived from studies of HDL biology in other inflammatory conditions, including post-surgical acute-phase responses, chronic metabolic diseases, and allergic airway inflammation. While extrapolation from these contexts has been informative, validation of these concepts in MPP-specific models represents an important direction for future research.

## 4 Receptor-mediated mechanisms of SAA-driven immunopathology

### 4.1 FPR2: neutrophil chemotaxis and NETs formation

**Figure 2:**
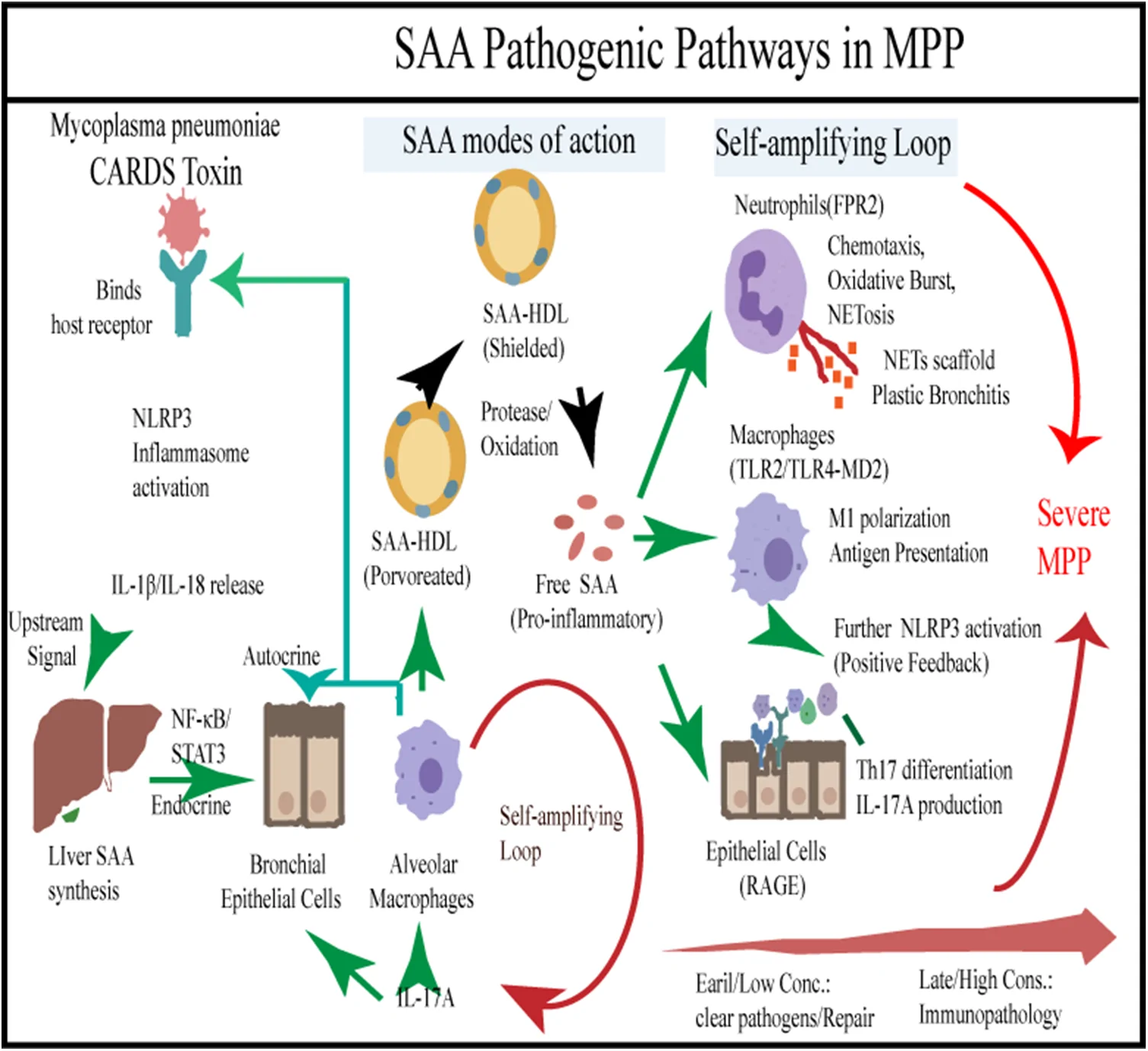
Proposed SAA-mediated immunopathogenic pathways in Mycoplasma pneumoniae pneumonia (MPP). The Community-Acquired Respiratory Distress Syndrome (CARDS) toxin binds to surfactant protein A (SP-A) and annexin A2 on airway epithelial cells, is internalized, and activates the NLR family pyrin domain containing 3 (NLRP3) inflammasome. This leads to caspase-1-dependent maturation and release of interleukin-1*β* (IL-1*β*), which stimulates hepatic and local pulmonary production of serum amyloid A (SAA). Free SAA engages multiple receptors: **(i)** formyl peptide receptor 2 (FPR2) on neutrophils, triggering chemotaxis, oxidative burst, and neutrophil extracellular trap (NET) formation; (ii) Toll-like receptor 4 (TLR4)/myeloid differentiation factor 2 (MD-2) on macrophages, promoting M1 polarization and further NLRP3 activation; and (iii) receptor for advanced glycation end products (RAGE) on epithelial cells, sustaining nuclear factor kappa B (NF-*κ*B) signaling. These receptor interactions establish a self-amplifying inflammatory loop. SAA-induced NETs contribute to mucus plugs and bronchial casts characteristic of plastic bronchitis. Dashed arrows indicate positive feedback loops. This figure integrates mechanisms described in (8, 10, 11, 47, 52, 55, 58).

Formyl peptide receptor 2 (FPR2, also known as FPRL1 or ALX) is a G-protein–coupled receptor (Figure 2).

primarily serving as the SAA receptor on neutrophils, monocytes, and macrophages (47). When SAA binds to FPR2, it triggers G*α*i-dependent signaling, leading to intracellular calcium release, extracellular signal-regulated kinase 1/2 (ERK1/2) phosphorylation, and activation of the phosphoinositide 3-kinase–protein kinase B (PI3K–Akt) pathway (48). These signaling events facilitate neutrophil chemotaxis, guiding their migration to *M. pneumoniae* infection sites.

In MPP, SAA signaling through FPR2 leads to several immunopathological effects. Initially, SAA activates nicotinamide adenine dinucleotide phosphate (NADPH) oxidase in neutrophils, causing an oxidative burst that produces reactive oxygen species (ROS), which in turn contribute to epithelial damage (49). Additionally, SAA significantly promotes the formation of NETs. This process involves peptidylarginine deiminase 4 (PAD4)-mediated histone citrullination, chromatin decondensation, and nuclear membrane rupture (50, 51). NETs, which consist of decondensed chromatin adorned with histones, myeloperoxidase, and neutrophil elastase, are found in abundance in airway secretions of patients with severe MPP and are associated with the development of plastic bronchitis (52).

SAA-induced NETs provide a proposed mechanistic framework for bronchial cast formation, a key feature of severe MPP, by creating a DNA and histone scaffold that captures mucus, fibrin, and cellular debris (53). This viscous DNA-histone matrix, along with interleukin-17 (IL-17)-driven MUC5AC overproduction, leads to the airway obstruction typical of plastic bronchitis. However, direct causal demonstration of the SAA-NETs-mucus plug pathway in human MPP-associated plastic bronchitis has not been established; current evidence derives primarily from *in vitro* NETosis assays and animal models. Additionally, components of NETs, especially histones and myeloperoxidase, are directly toxic to airway epithelial cells, exacerbating tissue damage.

Recent research by Yaeger et al. (54) has shown that ALX/FPR2 plays a role in SAA-induced lung neutrophil recruitment after acute ozone exposure. This finding underscores the importance of this pathway in pulmonary inflammation, even in the absence of infectious triggers.

### 4.2 TLR4/MD-2: macrophage M1 polarization and NLRP3 activation

Toll-like receptor 4 (TLR4), together with the the co-receptor myeloid differentiation factor 2 (MD-2), has been identified as a second important receptor for SAA (55). The interaction between SAA and the TLR4/MD-2 complex triggers both myeloid differentiation primary response 88 (MyD88)-dependent and TIR-domain-containing adapter-inducing interferon-*β* (TRIF)-dependent signaling pathways, resulting in the activation of NF-*κ*B and interferon regulatory factor 3 (IRF3) (56). In alveolar macrophages and recruited monocytes, SAA-TLR4 signaling encourages M1 polarization. This polarization is marked by increased expression of inducible nitric oxide synthase (iNOS) and elevated production of pro-inflammatory cytokines such as TNF-*α*, IL-6, interleukin-12 (IL-12), and interleukin-23 (IL-23), while simultaneously suppressing anti-inflammatory cytokines interleukin-10 (IL-10) and transforming growth factor-beta (TGF-*β*) (57).

SAA-TLR4 signaling notably triggers the NLRP3 inflammasome through a two-step mechanism. The priming step (Signal 1) involves NF-*κ*B-dependent upregulation of NLRP3 and pro-IL-1*β*. The activation step (Signal 2) occurs via potassium efflux, mitochondrial ROS production, and lysosomal destabilization (58, 59). This process forms a proposed self-amplifying loop: the CARDS toxin activates NLRP3, leading to IL-1*β* secretion, which induces SAA. SAA then re-engages TLR4, further activating NLRP3. However, the complete integrated loop has yet to be experimentally verified in a single model. In MPP, TLR4 is activated by both *M. pneumoniae* lipoproteins (pathogen-derived ligands) and SAA (host-derived ligand), accounting for the intense and persistent inflammation observed in RMPP (60).

The effects of SAA on macrophage polarization vary with concentration. At low concentrations (<50 mg/L), SAA promotes M2-like polarization, characterized by IL-10 production and tissue repair. Conversely, at high concentrations (>100 mg/L), SAA induces M1 polarization, leading to tissue-damaging inflammatory responses. This bimodal response might elucidate the biphasic nature of MPP. Initially, elevated SAA levels aid in pathogen clearance, but prolonged elevation can result in immunopathology (61). These concentration-dependent effects have been characterized primarily in THP-1 human macrophage cell lines and human aortic endothelial cells *in vitro*, as well as in murine sepsis models; species-specific differences in TLR4 signaling may limit direct translation to human MPP.

### 4.3 Receptor synergy: SAA as a molecular hub

SAA functions as a “molecular hub,” interacting with multiple receptors either simultaneously or sequentially to integrate various innate immune pathways (62). Notably, FPR2 and TLR4 exhibit functional synergy: FPR2-mediated calcium signaling primes TLR4 responses, while TLR4-induced NF-*κ*B activation enhances FPR2 expression. TLR2, which recognizes the amphipathic *α*-helical domains of SAA, plays a role in macrophage activation, especially in chronic inflammatory settings. RAGE identifies SAA fibrillar aggregates, contributing to prolonged NF-*κ*B activation. Scavenger receptor class B type I (SR-BI) facilitates SAA-HDL uptake, potentially aiding cholesterol delivery to inflamed tissues. Additionally, the P2X7 receptor, activated by SAA-induced adenosine triphosphate (ATP) release, offers another pathway for NLRP3 activation (63).

The redundancy of these receptors holds significant therapeutic implications, suggesting that effective SAA blockade might necessitate targeting multiple pathways. The influence of each receptor differs depending on the disease context. For instance, in MPP, FPR2 and TLR4 are predominant, whereas in Chronic Obstructive Pulmonary Disease (COPD)/emphysema, TLR2 is more influential (64). Understanding the specific contributions of these receptors in various respiratory diseases is important for the development of targeted therapies.

### 4.4 SAA as a soluble pattern recognition receptor

Emerging evidence suggests that, in addition to its function as a receptor ligand, SAA acts as a soluble PRR by directly binding to pathogen surface structures (65). SAA has been observed to attach to outer membrane protein A (OmpA) of Gram-negative bacteria, which enhances neutrophil phagocytosis and aids in bacterial clearance. In tuberculosis models, SAA interacts with mycobacterial membrane proteins such as AtpA, ABC transporter, and EspB. This interaction promotes macrophage uptake and simultaneously enhances antigen presentation through the upregulation of major histocompatibility complex (MHC) class I and II (66, 67).

SAA demonstrates direct antimicrobial properties. It has been shown to inhibit *Escherichia coli* growth at concentrations exceeding 50 mg/L. Additionally, SAA induces the secretion of MUC3 mucin in intestinal epithelial cells, which reduces the adhesion of pathogenic bacteria. Its amphipathic structure enables direct interaction with bacterial membranes, leading to their permeabilization (68). These opsonizing and antimicrobial functions might be significant in MPP, where SAA could potentially enhance the clearance of *M. pneumoniae* by alveolar macrophages. However, this hypothesis requires further investigation. The lack of a cell wall and reliance on host cholesterol may make it particularly vulnerable to SAA-mediated membrane disruption. Despite this vulnerability, *M. pneumoniae* may have developed evasion strategies, such as cholesterol scavenging through the P116 protein and biofilm formation, to counteract the effects of SAA (65).

## 5 Clinical applications of SAA in Mycoplasma pneumoniae pneumonia

### 5.1 Diagnostic value

#### 5.1.1 Diagnosis of Mycoplasma pneumoniae infection

SAA has shown notable diagnostic performance for MPP across various independent cohorts. For assessing MPP severity, Chen et al. reported that SAA distinguished severe from mild MPP with an AUC of 0.921 (cut-off 186.18 mg/L, sensitivity 92.18%, specificity 82.43%, *P* < 0.05) (70). For the diagnosis of MPP in children, Li et al. reported that SAA alone achieved a sensitivity of 91.43%, specificity of 97.14%, positive predictive value of 96.97%, and negative predictive value of 91.89% (69). Xian and Ding reported that SAA distinguished MPP from healthy controls with an AUC of 0.942 (95% confidence interval [CI] 0.911–0.973, cut-off 37.555 mg/L, sensitivity 81.2%, specificity 90.5%) (104). SAA has been reported to show superior diagnostic performance compared to CRP in several clinical studies, attributable to its rapid induction kinetics (71). Compared with conventional inflammatory biomarkers such as CRP and PCT, SAA exhibits more rapid induction after inflammatory stimulation and may provide additional information for early assessment of disease evolution; however, its limited specificity requires interpretation together with clinical findings and other biomarkers. Furthermore, optimal diagnostic cut-off values for SAA vary across study populations, and standardized thresholds for clinical use remain to be established. Table 1 summarizes the comparative characteristics of SAA and commonly used inflammatory biomarkers, highlighting their clinical utility and limitations in MPP and related respiratory diseases.

#### 5.1.2 Prediction of refractory Mycoplasma pneumoniae pneumonia

Ye et al. reported that acute-phase SAA predicted RMPP development with an AUC of 0.872 (cut-off 232.4 mg/mL, sensitivity 76.7%, specificity 89.13%) (103). Consistently, Ma et al. reported that serum SAA showed an AUC of 0.735 for distinguishing RMPP from non-RMPP MPP, with TNF-*α* and CARDS toxin showing AUCs of 0.711 and 0.705, respectively (16).

Combining SAA with the NLR and lactate dehydrogenase (LDH) has been reported to enhance predictive accuracy. Deng and Luo (72) identified SAA, NLR, and LDH as independent predictors of RMPP by multivariate logistic regression (OR = 1.009, 1.552, and 1.012, respectively; all *P* < 0.05). Individually, SAA showed an AUC of 0.736 (sensitivity 82.10%, specificity 59.60%), NLR an AUC of 0.752 (sensitivity 73.10%, specificity 69.20%), and LDH an AUC of 0.766 (sensitivity 51.30%, specificity 86.50%). The combined SAA + NLR + LDH model achieved an AUC of 0.865 (sensitivity 73.10%, specificity 84.00%) (72). Beyond predicting the development of RMPP, recent studies have also explored prognostic stratification among children who have already developed RMPP. Additionally, Chang et al. (73) created a prediction model for adverse prognosis in children with RMPP using serum SAA/CRP and retinol-binding protein (RBP) levels, achieving an AUC of 0.920, with a sensitivity of 0.817 and specificity of 0.899. In this model, elevated SAA/CRP was an independent risk factor for poor prognosis (OR = 2.908, 95% CI 1.589–5.323), along with prolonged fever (OR = 1.420, 95% CI 1.085–1.860), severe RMPP (OR = 6.147, 95% CI 2.346–16.111), and extended oxygen therapy (OR = 2.187, 95% CI 1.421–3.364), while higher RBP was protective (73). These combined-marker models are derive from single-center studies and require validation in independent, multicenter cohorts. The variability in reported AUC values for SAA across these studies (0.735−0.872) may reflect differences in RMPP definitions, sample sizes, timing of SAA measurement, and the specific statistical models employed. Standardization of RMPP diagnostic criteria and SAA sampling protocols would facilitate more meaningful cross-study comparisons.

#### 5.1.3 Biological basis of SAA as a diagnostic marker

The diagnostic value of SAA is rooted in its biological characteristics. First, its rapid induction within 4-6 hours allows for early detection, preceding the elevation of CRP. Second, its large dynamic range, with up to a 1000-fold increase, allows severity stratification. Lastly, the correlation between SAA levels and CARDS toxin-mediated pathology is consistent with the hypothesis that SAA reflects disease-specific inflammatory pathways. However, it should be noted that SAA is an acute-phase reactant elevated in a broad range of conditions including bacterial sepsis, trauma, and autoimmune disease; its diagnostic specificity for MPP depends on the clinical context and should be interpreted alongside other markers.

#### 5.1.4 Limitations and challenges in clinical implementation

The diagnostic metrics reported for SAA in MPP must be interpreted with awareness of several constraints that affect its translation into routine clinical practice Table 2.

**Table 2:** Comparison of SAA with commonly used inflammatory biomarkers in MPP and related respiratory diseases.

Biomarker	Diagnostic role	Kinetics	Advantages	Limitations
SAA	MPP diagnosis and RMPP prediction	Rapid induction (4–6 h)	High acute-phase response; early elevation	Limited specificity
CRP	Inflammation assessment	24–48 h after SAA	Widely available; low cost	Less sensitive in early phase
PCT	Bacterial infection and severity indicator	Early but infection-dependent	Helps distinguish bacterial from viral inflammation	Limited data specifically in MPP
LDH	Tissue injury and severity marker	Reflects tissue damage	Associated with severe MPP and RMPP	Not specific; elevated in many conditions
IL-6	Cytokine activity marker	Early immune activation	Mechanistic relevance to SAA pathway	Limited clinical availability; high cost

Disease specificity is the most fundamental limitation. SAA responds to diverse inflammatory, infectious, and tissue-injury stimuli without discriminating among etiologies. An elevated SAA level alone cannot reliably distinguish MPP from bacterial pneumonia, viral bronchiolitis, or non-infectious inflammatory conditions such as Kawasaki disease or systemic juvenile idiopathic arthritis. This limitation is particularly relevant in severe MPP, where bacterial superinfection occurs in 10–30% of cases and would produce concurrent SAA elevation through overlapping cytokine pathways.

Systemic inflammatory conditions confound interpretation. Obesity, metabolic syndrome, type 2 diabetes, and chronic cardiovascular disease are each associated with chronically elevated baseline SAA levels, typically 10–40 mg/L compared with <10 mg/L in healthy individuals (46). In such patients, the fold-increase attributable to acute MPP may be blunted, or conversely, baseline elevation may be misattributed to infection. The pediatric populations in which MPP is most prevalent generally have lower rates of these comorbidities, but the increasing prevalence of childhood obesity in regions with high MPP incidence warrants consideration.

Assay variability complicates cross-study comparison and clinical decision-making. As detailed in Section 2.2, different commercial platforms—enzyme-linked immunosorbent assay, immunonephelometry, latex-enhanced immunoturbidimetry, and gold-labeled quantitative immunoassay—employ distinct antibodies, calibrators, and detection chemistries (36). Reported cutoffs for “elevated” SAA range from >10 mg/L to >100 mg/L depending on the assay and clinical context.

The AUC of 0.942 reported by Xian and Ding (104) was generated using a specific platform with a cutoff of 37.555 mg/L; whether this threshold performs equivalently across assays is unknown. International reference standards and MPP-specific reference ranges have not been established.

The rapid kinetics of SAA, while advantageous for early detection, also create interpretive challenges. A single measurement captures only one time point in a dynamic response that peaks, plateaus, and declines at rates influenced by treatment response, complication development, and individual variation in hepatic synthetic capacity.

Serial monitoring is more informative than isolated values, yet standardized protocols for timing and frequency of SAA measurement in MPP have not been defined.

Finally, the demographic concentration of clinical studies limits generalizability. The diagnostic and prognostic AUC values cited derive predominantly from single-center observational studies in Chinese pediatric cohorts (69–75, 104). Whether these performance characteristics extend to other ethnic populations, adult patients, or healthcare settings with different epidemiological patterns of *M. pneumoniae* macrolide resistance remains to be determined. Multicenter validation with standardized assays would substantially strengthen the evidence base for clinical implementation.

### 5.2 Dynamic monitoring: treatment response and prognosis prediction

In observational clinical studies, serial measurements of SAA offer insights into treatment response and prognosis. A reduction in SAA levels by more than 50% within 72 hours of starting treatment has been associated with favorable clinical outcomes (74). Nie and Yang (75) reported that an elevated SAA/high-sensitivity C-reactive protein (hs-CRP) ratio was associated with poor treatment response in MPP (AUC=0.718, 95% CI 0.601–0.835; cut-off 6.04, sensitivity 68.8%, specificity 87.2%). Combined detection of SAA/hs-CRP, NLR, and PCT improved prognostic accuracy (AUC=0.825, 95% CI 0.737–0.914, sensitivity 83.3%, specificity 71.9%) (75). If SAA levels rise again after an initial decrease, this may suggest bacterial superinfection or complicationssuch as pleural effusion (76).

The >50% reduction threshold derives from observational studies and requires validation in prospective cohorts before it can guide clinical decision-making.

Ma et al. demonstrated that serum and BALF levels of SAA, CARDS TX, and TNF-*α* were significantly elevated in children with RMPP compared with non-RMPP MPP. Among these biomarkers, serum SAA showed the highest individual predictive performance (AUC=0.735), followed by TNF-*α* (AUC=0.711) and CARDS toxin (AUC=0.705) (16). These findings derive from a single-center study and require replication in independent cohorts.

### 5.3 Immune injury mechanisms and long-term sequelae

SAA has been implicated in MPP immunopathology through various effector mechanisms. *In vitro* studies have shown that SAA can enhance Th17 cell differentiation and increase interleukin-17 (IL-17) production (77). IL-17, in turn, stimulates airway epithelial cells to release chemokines such as CXCL8, CXCL1, and CXCL5, which attract neutrophils and create a self-perpetuating inflammatory cycle (78). In human pediatric studies, Interleukin-17A (IL-17A) levels are significantly higher in children with RMPP compared to those with non-RMPP (79). Additionally, distinct pediatric MPP endotypes have been identified based on serum IL-17A and IL-6 profiles, highlighting the potential for personalized therapeutic approaches (80). The SAA-Th17-IL-17-chemokine cascade has been demonstrated in separate experimental systems; the complete pathway has not been validated in a single integrated model of MPP.

The formation of SAA-induced NETs serves as a framework for mucus plug development, which plays a role in the formation of bronchial casts in plastic bronchitis (52, 53). Li et al. (52) identified SAA ≥ 256.205 mg/L, length of stay ≥ 7.5 days, fever duration ≥ 6.5 days, CRP ≥ 13.11 mg/L, and ferritin ≥ 97.7 ng/mL as independent risk factors for airway mucus plugs in children with community-acquired RMPP. A combined diagnostic model achieved an AUC of 0.920 (95% CI 0.856–0.983, *P* < 0.001) (52). Implementing early bronchoscopic intervention alongside anti-inflammatory therapy targeting the SAA-NETs axis represents a potential therapeutic strategy warranting clinical investigation. However, targeting the SAA-NETs axis as a therapeutic strategy remains a hypothesis; no clinical trial has demonstrated improved outcomes with this approach in MPP.

As reviewed by Ji et al., SAA exhibits pro-coagulant properties by promoting tissue factor expression on monocytes and endothelial cells, as well as enhancing platelet adhesion and aggregation (82, 83). In severe MPP, pulmonary microthrombosis can lead to ventilation-perfusion mismatch and hypoxemia. Elevated SAA levels have been reported to increase platelet adhesion in patients with coronavirus disease 2019 (COVID-19) (81). whether a similar mechanism operates in severe MPP remains to be investigated. Direct evidence for SAA-driven microthrombosis in the lungs of patients with MPP is limited and derives primarily from *in vitro* studies and observations in other respiratory diseases. When these acute inflammatory mechanisms persist or fail to resolve, they may contribute to long-term respiratory complications, including bronchiolitis obliterans and pulmonary fibrosis.

#### 5.3.1 Long-term sequelae: bronchiolitis obliterans and pulmonary fibrosis

Emerging evidence indicates that SAA may play a role in chronic airway diseases following MPP. In patients who develop bronchiolitis obliterans after severe MPP, persistent elevation of SAA has been associated with the severity of airflow obstruction, as reviewed by Papareddy et al. (83). Several potential mechanisms are proposed: sustained activation of group 3 innate lymphoid cells (ILC3) leading to increased IL-17 production and neutrophilic inflammation has been suggested as a contributing pathway; SAA-induced TGF-*β* signaling, which may trigger epithelial-mesenchymal transition and airway fibrosis (84); and the chronic release of NETs that exacerbates airway inflammation and remodeling (85). The evidence for these mechanisms derives primarily from experimental models and clinical correlation studies; direct causal demonstration in post-MPP bronchiolitis obliterans is lacking.

The generalizability of these findings is further constrained by the demographic concentration of clinical studies. As noted in Section 5.1.4, the majority of SAA studies in MPP derive from single-center Chinese pediatric cohorts (69–77). Whether SAA thresholds for severity prediction, dynamic response patterns during treatment, and associations with long-term sequelae apply to other populations requires prospective multicenter validation.

In a mouse model of bleomycin-induced pulmonary fibrosis, Yang et al. (84) recently showed that SAA3 exacerbates pulmonary fibrosis by promoting interleukin-36*α* expression through Krüppel-like factor 6. This discovery offers potential mechanistic insights into the profibrotic role of SAA. Whether these findings extend to post-infectious fibrosis after MPP remains to be determined. To confirm the causal link between persistent SAA levels and chronic airway disease post-MPP, long-term studies are essential. These studies should include serial SAA measurements, pulmonary function tests, and high-resolution CT imaging. Notably, SAA3 is a pseudogene in humans, and the relevance of murine SAA3 findings to human post-infectious fibrosis requires cautious interpretation.

It should be noted that the majority of clinical SAA studies in MPP discussed in this section originate from Chinese pediatric cohorts (primarily single-center observational studies with limited external validation in diverse populations) (69–77). While these studies provide valuable evidence for the clinical utility of SAA in MPP, their generalizability to other populations, healthcare systems, and epidemiological contexts requires validation. Multicenter international studies with diverse ethnic populations are needed to confirm the diagnostic and prognostic thresholds reported here.

## 6 SAA in related respiratory diseases

### 6.1 Chronic airway diseases: asthma and COPD

**Figure 3:**
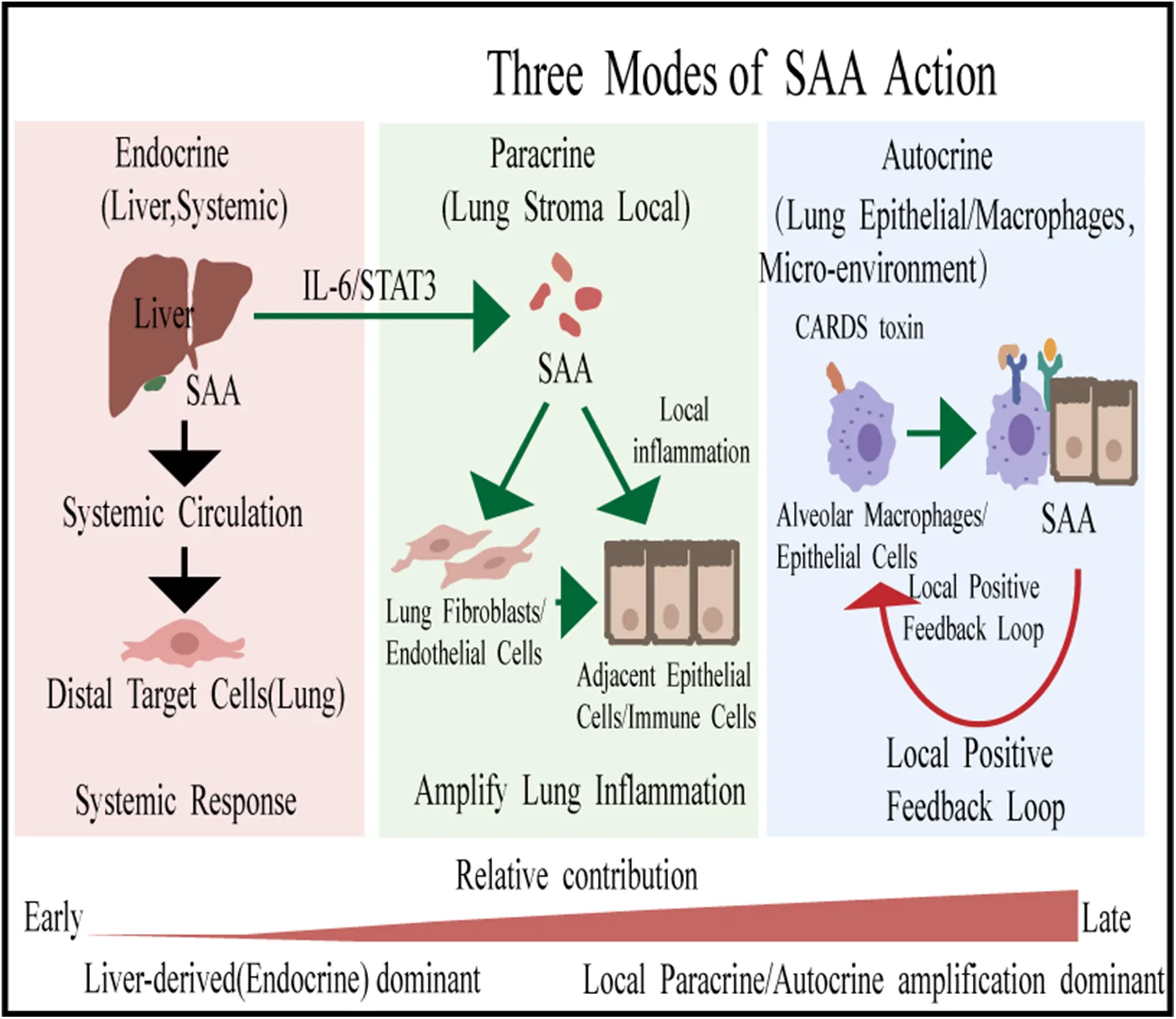
Three proposed modes of SAA action in MPP. **(A)** Endocrine mode: hepatic SAA enters systemic circulation and reaches the lungs. **(B)** Paracrine mode: pulmonary stromal cells (fibroblasts and endothelial cells) produce SAA that acts on adjacent epithelial and immune cells. **(C)** Autocrine mode: alveolar macrophages and bronchial epithelial cells synthesize SAA, which acts on the same cell types. The relative contribution of each mode varies with disease stage; early MPP is dominated by endocrine SAA, whereas severe or progressive MPP involves increased paracrine and autocrine amplification. This conceptual model synthesizes evidence from (16, 28, 29).

In a clinical study of children with asthma and MPP, those infected with *M. pneumoniae* showed significantly higher SAA levels than non-asthmatic peers, which correlate with IgE, “interleukin-4 (IL-4)”/interferon-*γ* (IFN-*γ*) ratios, TGF-*β*1, and lung function metrics (86). Xiao and Hou (86) highlighted that combining SAA and IgM provides strong diagnostic value for asthma in children with MPP. Patients with high SAA levels (SAA ≥108.8 mg/mL) alsoexhibit elevated CRP, IL-6, and TNF-*α*, alongside a higher incidence of obesity and severe asthma (46). Mechanistically, *in vitro*, HDL from these patients with high SAA levels has been shown to trigger monocyte secretion of IL-6, IL-1*β*, and TNF-*α* via the FPR2/ATP/P2X7 pathway, worsening allergen-induced neutrophilic airway inflammation and goblet cell metaplasia. (Figure 3).

Recent studies have identified a proposed mechanism in COPD and emphysema where SAA contributes to alveolar destruction via the neutrophil- ILC3 axis (64). Pulmonary SAA3 levels are notably elevated without corresponding hepatic changes, primarily produced by monocytes and dendritic cells. In patients with emphysema and COPD, sputum SAA1 levels and ILC3 frequency are higher compared to those in non-emphysema controls. Mechanistically, SAA activates neutrophils through FPR2/TLR2/TLR4, leading to IL-1*β* secretion. This IL-1*β* promotes ILC3 proliferation andIL-17A production. Subsequently, ILC3s stimulate neutrophils to secrete matrix metalloproteinase-12 (MMP-12) via IL-17A, creating a proposed SAA-neutrophil-IL-1*β*-ILC3-neutrophil-MMP-12 positive feedback loop that results in lung tissue destruction.

Notably, depleting ILC3s improves emphysema without affecting neutrophil infiltration, highlighting ILC3s as key emphysematogenic cells in animal models (105). As noted above, SAA3 is a pseudogene in humans, and the translational relevance of these murine findings requires cautious interpretation. However, each step of this proposed feedback loop has been characterized in separate experimental systems, and the complete loop has not been experimentally validated in human COPD.

These findings may have implications for understanding post-MPP chronic airway disease. The SAA-neutrophil-ILC3 pathway identified in COPD parallels the proposed CARDS toxin-NLRP3-IL-1*β*-SAA loop in MPP (see Section 5.3). Furthermore, the important role of ILC3s in emphysema suggests a potential link between post-MPP chronic airway lesions, such as bronchiolitis obliterans, and the ongoing activation of the SAA-ILC3-IL-17A axis.

### 6.2 Interstitial lung diseases: idiopathic pulmonary fibrosis

In clinical studies, SAA has been studied as a potential biomarker for idiopathic pulmonary fibrosis (IPF), a progressive interstitial lung disease marked by chronic inflammation, abnormal wound healing, and irreversible fibrosis. Research indicates that IPF patients exhibit significantly elevated serum SAA levels compared to healthy individuals, with a diagnostic threshold of 6300 ng/mL offering 71% sensitivity and 85% specificity (87). Additionally, Vietri et al. (88) found that serum SAA levels in patients with IPF are notably higher than those in patients with other interstitial lung diseases, with levels exceeding 4057.8 µg/mL linked to a poor prognosis. The marked difference in reported thresholds likely reflects differences in assay methodology, patient populations, and disease severity between studies.

SAA levels in IPF are inversely related to predicted forced vital capacity (FVC%) and independently forecast lung function decline (92). Bergantini et al. (89) found that combining SAA with KL-6 yields an AUC of 0.81 for differentiating IPF from chronic hypersensitivity pneumonitis and sarcoidosis. Additionally, SAA has merged as a promising biomarker for early IPF diagnosis, as highlighted in a systematic review (90).

The observed negative correlation between SAA and FVC% in IPF aligns with similar findings in MPP indicating that SAA might serve as a broadly applicable marker for assessing the severity of lung parenchymal damage across various lung injury diseases. However, it is important to note the distinction between these conditions: IPF is a chronic, progressive disease where sustained SAA elevation signals irreversible fibrosis. In contrast, MPP is an acute infectious disease where SAA levels may indicate reversible inflammatory injury. This temporal difference is important for therapeutic strategies.

### 6.3 Acute infectious pneumonias: COVID-19

In observational studies, over 83% of patients with COVID-19 exhibit elevated SAA levels, which strongly correlate with disease severity (92, 93). Receiver operating characteristic (ROC) analysis reveals that SAA achieves an AUC of 0.818 to 0.947 in predicting COVID-19 severity. An SAA level exceeding 157.9 mg/L is a reported threshold for indicating disease progression (91).Additionally, deceased patients have significantly higher SAA levels compared to survivors, with medians of 740 mg/L and 487.5 mg/L, respectively (93).

SAA has been implicated in COVID-19 hyperinflammatory syndrome through several mechanisms. It induces pro-inflammatory cytokines and chemokines, stimulates neutrophil oxidative burst at high concentrations, and activates the NLRP3 inflammasome *in vitro* via the P2X7 receptor and cathepsin B-sensitive pathways. Additionally, SAA promotes the migration of monocytes, neutrophils, and T cells (59, 94, 95).

Comparing MPP and COVID-19 reveals both shared patterns and distinct disease-specific characteristics. Both are respiratory infections where SAA plays diagnostic and prognostic roles. COVID-19 typically results in significantly higher SAA levels (hundreds to thousands of mg/L), which are linked to acute respiratory distress syndrome (ARDS) and multi-organ failure. In contrast, MPP leads to moderate SAA increases (tens to hundreds mg/L),mainly associated with pulmonary complications. A critical difference lies in the inflammatory pathways. In COVID-19, SAA activates the NLRP3 inflammasome, promoting the secretion ofinterleukin-1*β* (IL-1*β*) (SAA→NLRP3→IL-1*β*). Conversely, in MPP, the CARDS toxin activatesNLRP3, which subsequently induces SAA production (CARDS toxin→NLRP3→IL-1*β*→SAA;a hypothesized pathway; see Section 5.3). A tentative mechanistic distinction. emerges from available evidence: in COVID-19, SAA functions primarily as an amplifier of inflammation, whereas in MPP, SAA is positioned as both a product and a contributor to the inflammatory response. This framework integrates separate findings from each disease but awaits direct comparative experimental validation.

### 6.4 Acute lung injury and ARDS

In murine models of sepsis-associated acute lung injury, SAA demonstrates context-dependent roles. Exosomal SAA1 from bone marrow mesenchymal stromal cells can mitigate lung injury induced by cecal ligation and puncture or LPS, lowering endotoxin, TNF-*α*, and IL-6 levels (64). In contrast, silencing SAA1 reduces LPS-induced lung injury, edema, and neutrophil infiltration (95). This suggests that SAA family members can have both anti-inflammatory and pro-inflammatory effects, influenced by their concentration, source, and receptor environment.

In a retrospective study involving 110 patients with ARDS, researchers found that serum levels of SAA, NLR, and PCT significantly increased with disease severity (96). Deceased patients exhibited SAA levels of 117.19 ± 16.01 mg/L, notably higher than the 104.54 ± 15.65 mg/L observed in survivors. SAA demonstrated an AUC of 0.762 for mortality prediction, comparable to that of NLR (0.769) but lower than that of PCT (0.911). The combined predictive value of SAA with NLR and PCT in ARDS is conceptually analogous to the SAA + NLR + LDH strategy proposed for RMPP prediction. Additionally, the prognostic AUC of SAA in ARDS (0.762) aligns with its predictive range for RMPP (0.736-0.872), suggesting that SAA-based prediction models may have comparable prognostic performance across different severe lung injuries. However, these observations derive from different patient populations with distinct disease etiologies, and their generalizability requires validation in disease-specific cohorts.

## 7 Cellular sources and modes of SAA action

### 7.1 Hepatic production and systemic regulation

**Figure 4:**
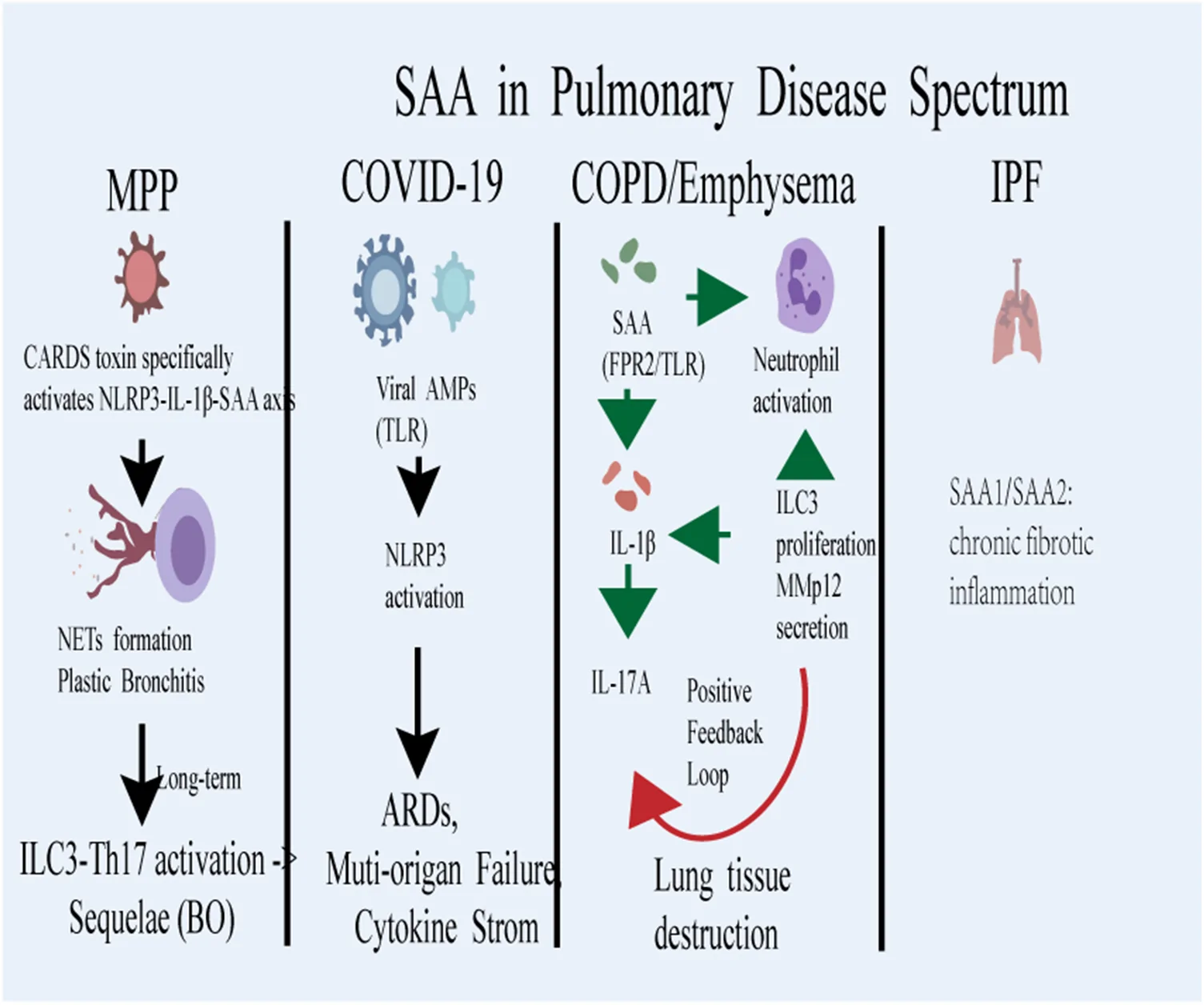
Role of SAA across pulmonary diseases. Shared pathogenic mechanisms (center) include NLRP3 inflammasome activation, neutrophil recruitment, cytokine and chemokine induction, and NET formation. Disease-specific characteristics (peripheral): MPP involves CARDS toxin-driven SAA induction via IL-1*β*; COVID-19 features viral pathogen-associated molecular patterns (PAMPs) activating TLR3/retinoic acid-inducible gene I (RIG-I) pathways; asthma is characterized by allergen-driven T helper 2 (Th2)/T helper 17 (Th17) skewing; COPD/emphysema involves the SAA-group 3 innate lymphoid cell (ILC3)-matrix metalloproteinase-12 (MMP-12) axis; and idiopathic pulmonary fibrosis (IPF) is marked by chronic fibrotic remodeling. The dual functionality of SAA—protective at low concentrations through opsonization and tissue repair, pathogenic at elevated levels via receptor-mediated inflammatory amplification—is context-dependent and varies with disease stage and SAA concentration.

Hepatic SAA synthesis via STAT3/NF-*κ*B pathways is well-characterized. The liver is the main producer of circulating SAA during acute-phase responses, contributing to over 90% of the increase in serum SAA levels (102). Hepatic synthesis of SAA is controlled by systemic cytokines such as IL-6, IL-1*β*, and TNF-*α*, primarily through STAT3 and NF-*κ*B signaling pathways. Among these, IL-6 is the primary inducer, activating STAT3 at the SAA promoter (26). In cases of MPP, systemic SAA levels are linked to the duration of fever, elevated inflammatory markers, and extrapulmonary complications (4). (Figure 4).

### 7.2 Extrahepatic SAA production: adipose tissue and the pulmonary microenvironment

In clinical and animal studies, significant extrahepatic production of SAA has been observed in adipose tissue. In mice, SAA3, and in humans, SAA1/SAA2, contribute to both local and systemic inflammation associated with obesity (46). In the lungs, SAA is synthesized by bronchial epithelial cells, alveolar macrophages, and pulmonary fibroblasts in response to direct microbial stimulation (28). This local production has been proposed to establish paracrine/autocrine amplification loops that can sustain pulmonary inflammation independently of systemic SAA levels. Consequently, in a pediatric MPP cohort (*n* = 132), BALF SAA levels were significantly higher in children with refractory MPP compared to non-refractory cases (*P* < 0.05), with an AUC of 0.735 for predicting refractory disease, suggesting that pulmonary SAA may reflect local disease severity more directly than systemic SAA (16). Correlation coefficients between BALF SAA and quantitative lung injury indices were not reported in the original study (16); available data are limited to group comparisons and AUC-based predictive performance.

### 7.3 Three modes of SAA action: endocrine, paracrine, and autocrine

SAA operates in MPP through three distinct modes based on its source and range of action: (1) endocrine: SAA produced by the liver enters the bloodstream, reaching the lungs to act on distant targets; (2) paracrine: SAA generated by lung stromal cells affects nearby epithelial and immune cells within the pulmonary microenvironment; and (3) autocrine: SAA synthesized by alveolar macrophages or epithelial cells acts on the same cells, potentially contributing to their activated state. The contribution of each mode shifts with the disease stage—early MPP likely involves primarily endocrine SAA, while severe or progressive MPP may involve greater paracrine/autocrine amplification. This three-mode framework represents a proposed conceptual model synthesizing current evidence on SAA action in MPP.

## 8 Therapeutic targeting of SAA-mediated immunopathology

### 8.1 Current strategies: corticosteroids and indirect SAA suppression

In clinical studies, corticosteroids are a cornerstone of adjunctive therapy for severe MPP due to their broad anti-inflammatory effects, including the suppression of SAA production (97). They achieve this by inhibiting the transcriptional activities of NF-*κ*B and AP-1, which in turn reduces the production of IL-6 and IL-1*β*, thereby indirectly decreasing hepatic SAA synthesis. Shen et al. (98) investigated the timing of glucocorticoid therapy in children with *M. pneumoniae* pneumonia who had 23S rRNA domain V mutations. Their study found that initiating corticosteroid treatment within 72 hours of fever onset was linked to quicker fever resolution and a shorter hospital stay.

The non-specific immunosuppression caused by corticosteroids raises concerns about secondary infections, delayed pathogen clearance, and adrenal suppression (99). The timing of corticosteroid initiation is an important consideration. Early administration, within 48-72 hours of RMPP diagnosis, seems more effective than delayed treatment. This early intervention may interrupt the SAA amplification loop before severe lung injury develops. However, the optimal dosing and duration of corticosteroid treatment continue to be debated.

### 8.2 Emerging targets: FPR2 antagonists, NLRP3 inhibitors, and anti-IL-17a therapy

Currently, all strategies targeting the SAA pathway for MPP remain in preclinical investigation; no agent has entered clinical trials for this indication, and none are approved for any inflammatory lung disease. FPR2 antagonists, including WRW4 and BOC-2, have demonstrated *in vitro* blockade of SAA-induced neutrophil chemotaxis and NET formation (100), though their pharmacokinetic properties, safety profiles, and efficacy in MPP models have not been characterized. The 5-MP peptide, a synthetic derivative from the SAA receptor-binding domain, competitively inhibits SAA binding to FPR2 and TLR2/4 in biochemical assays and cell culture, thereby reducing neutrophil recruitment and cytokine production (101). Its stability in biological fluids, delivery to the lung, and therapeutic index *in vivo* remain uncharacterized.

NLRP3 inflammasome inhibitors, such as MCC950, have shown anti-inflammatory effects in preclinical models of multiple diseases and theoretically could disrupt the proposed CARDS toxin-NLRP3-IL-1*β*-SAA amplification loop in MPP. However, MCC950, which reached early-phase clinical development for rheumatoid arthritis, was subsequently discontinued due to hepatotoxicity concerns attributed to off-target effects, and no NLRP3 inhibitor is currently approved for human use.and no NLRP3 inhibitor is currently approved for human use. The safety of NLRP3 blockade during active M. pneumoniae infection—where inflammasome-derived IL-18 contributes to protective Th1 responses—is unknown. Although early preclinical results have shown anti-inflammatory effects, clinical application is challenged by concerns about specificity, long-term safety, and the potential to compromise protective inflammasome functions.

Anti-IL-17A antibodies (secukinumab, ixekizumab) are approved for autoimmune indications but represent only a tentative extrapolation to MPP based on shared Th17 pathway involvement. No preclinical MPP model has tested these agents, and their immunosuppressive effects during active respiratory infection raise substantial safety concerns. Furthermore, IL-17A contributes to mucosal antifungal immunity; its blockade in children with pneumonia could predispose to opportunistic infections. In summary, SAA-targeted therapeutic strategies for MPP remain entirely experimental. No direct SAA inhibitor, receptor antagonist, or upstream pathway modulator has been tested in MPP-specific models, let alone human clinical trials. The therapeutic proposals in this section derive from mechanistic reasoning and analogy to other diseases rather than evidence of efficacy or safety in MPP.

### 8.3 The therapeutic window: preserving host defense while blocking injury

In the context of host defense, a key challenge in SAA-targeted therapy is determining the optimal timing. In the early stages of infection, SAA contributes to host defense through opsonization of pathogens, enhances phagocytosis, and aids in tissue repair (65). Blocking SAA too early could weaken host defenses and heighten the risk of secondary infections. On the other hand, prolonged SAA elevation can lead to chronic inflammation and tissue remodeling, as shown in a murine bleomycin model (84).

Based on corticosteroid timing data in pediatric MPP (98), the therapeutic window for SAA-targeted interventions has been proposed to occur during the transition from the acute to subacute phases of illness, specifically between days 5 and 7, when the pathogen load is managed but inflammation persists (98). However, this timing has not been validated for SAA-directed therapies and represents a hypothesis requiring prospective evaluation. Biomarker-guided therapy, which involves dynamic monitoring of SAA to identify patients who could benefit from targeted interventions, exemplifies a precision-medicine approach. We propose a three-stage framework: (1) during the early phase(days 1-3), focus on preserving SAA-mediated antimicrobial defense; (2) in the subacute phase (days 5-7), evaluate the SAA trajectory and initiate targeted anti-inflammatory therapy for patients with persistent elevation; and (3) in the chronic phase (beyond 2 weeks), monitor for SAA-driven fibrotic sequelae and consider anti-fibrotic strategies for patients with sustained elevation. This framework requires prospective clinical validation.

## 9 Conclusions and future directions

SAA has emerged as a central mediator in MPP pathogenesis based on converging evidence from human clinical studies, animal models, and *in vitro* experiments, although direct causal demonstration in integrated human MPP models remains incomplete. It serves not just as a biomarker but as a likely contributor to disease pathogenesis. It engages multiple receptors—FPR2, TLR2/4, and RAGE—to enhance the CARDS toxin-NLRP3-IL-1*β* inflammatory cascade, promote NETs formation, induce mucus plugging, and promote Th17 differentiation. Additionally, SAA may contribute to long-term airway remodeling and fibrosis. Understanding the dual role of SAA, which is protective at low concentrations through opsonization and tissue repair but pathogenic at elevated levels through receptor-mediated amplification, represents an important consideration for developing targeted therapies.

Several fundamental questions remain unanswered in the study of SAA. First, what are the structural determinants of SAA receptor specificity, and is it possible to design subtype-selective modulators that maintain protective functions while inhibiting harmful signaling? Additionally, how do systemic versus local sources of SAA quantitatively contribute to pulmonary inflammation at various stages of disease? An important question is why some patients experience extreme SAA elevations and severe disease, while others have only modest responses. Could genetic polymorphisms in SAA genes (SAA1, SAA2) or receptor genes (FPR2, TLR4) affect susceptibility? Finally, identifying the precise concentration thresholds and temporal transitions at which SAA shifts from a protective acute-phase reactant to a pathogenic mediator in MPP remains a challenge.

We propose that to address these questions effectively, an integrated approach is essential. This approach should combine structural biology for detailed receptor-ligand characterization, single-cell genomics to identify cellular sources and responses, longitudinal biomarker studies using standardized assays specific to SAA subtypes, and meticulously designed clinical trials for targeted interventions. The ultimate aim is to translate the biology of SAA into precision therapies that selectively inhibit pathogenic SAA signaling while maintaining its protective acute-phase functions. This challenge pertains not only to MPP but also to the broader realm of inflammatory lung diseases.

In situating MPP within the range of SAA-associated pulmonary diseases, it is important to recognize both the shared pathways and unique characteristics that set it apart from other conditions like viral pneumonia, chronic airway disease, and interstitial lung disease. Common pathways include NLRP3 activation, neutrophil recruitment, and cytokine induction. However, MPP is distinguished by specific features such as the CARDS toxin, local pulmonary synthesis, and its association with NETs and plastic bronchitis. The recently identified neutrophil-ILC3 axis in COPD and emphysema may serve as a mechanistic link between acute MPP and subsequent chronic airway complications. This hypothesis merits focused research. Developing MPP-specific animal models with inducible, cell-type-specific SAA modulation is essential for unraveling these mechanisms and assessing potential therapies before they are applied clinically.
